# Ausgeprägte und disseminierte atypische Mykobakteriose der Haut unter Immunsuppression

**DOI:** 10.1111/ddg.70016

**Published:** 2026-06-04

**Authors:** Veronika Zenderowski, Laura Schreieder, Mark Berneburg, Dennis Niebel, Sebastian Haferkamp, Sigrid Karrer, Konstantin Drexler

**Affiliations:** ^1^ Klinik und Poliklinik für Dermatologie Universitätsklinikum Regensburg Regensburg Deutschland

**Keywords:** Atypische Mykobakteriose, Immunsuppression, TNF‐Alpha, Morbus Crohn, Atypical mycobacteriosis, immunosuppression, TNF‐alpha, Crohn's disease

Sehr geehrte Herausgeber,

 atypische Mykobakteriosen der Haut sind seltene Infektionen, die insbesondere bei immungeschwächten Patienten auftreten. ^1^ Ihr variables klinisches Bild erschwert die Diagnosestellung. Wir berichten über einen Patienten mit Morbus Crohn, der mit Adalimumab behandelt wurde und bei dem sich eine disseminierte Mycobacterium‐marinum‐Infektion entwickelte. Anfangs wurden die Hautveränderungen als mögliche Arzneimittelreaktion oder als kutane Manifestation des Morbus Crohn gedeutet. Dies verdeutlicht die diagnostische Herausforderung.

Ein weiterer Patient wurde aufgrund neu aufgetretener, schmerzhafter Hautveränderungen von der Rheumatologie zur dermatologischen Mitbeurteilung vorgestellt. Der Patient litt seit 13 Jahren an einem Morbus Crohn, der ursprünglich mit Methotrexat (MTX) behandelt wurde. Aufgrund der unzureichenden Wirksamkeit dieser Therapie wurde die Behandlung vor etwa einem Jahr auf Adalimumab umgestellt. Kurz darauf entwickelte der Patient erstmals schmerzhafte Hautveränderungen an den Händen. Aufgrund der unzureichenden Symptomkontrolle der Darmerkrankung erfolgte eine Umstellung der Therapie auf Infliximab, woraufhin sich der Hautbefund weiter verschlechterte. Auch die Behandlung mit Prednisolon führte zu einer zunehmenden Ausbreitung der Läsionen, die schließlich das gesamte Integument betrafen. Der Patient arbeitete in der Grünflächenpflege und betreute privat ein Aquarium, besetzt mit Kaltwasserfischen.

Die klinische Inspektion ergab am gesamten Integument disseminierte, scharf begrenzte, bis mehrere Zentimeter große livid‐erythematöse Nodi. Daneben traten stecknadelkopfgroße erythematöse Papeln sowie mehrere Zentimeter durchmessende Plaques auf, von denen einzelne deutlich überwärmt waren (Abbildung 1a–c). An Armen und Beinen zeigte sich zudem ein sporotrichoides Ausbreitungsmuster mit einer Anordnung der Effloreszenzen entlang der Lymphbahnen.

**ABBILDUNG 1:**
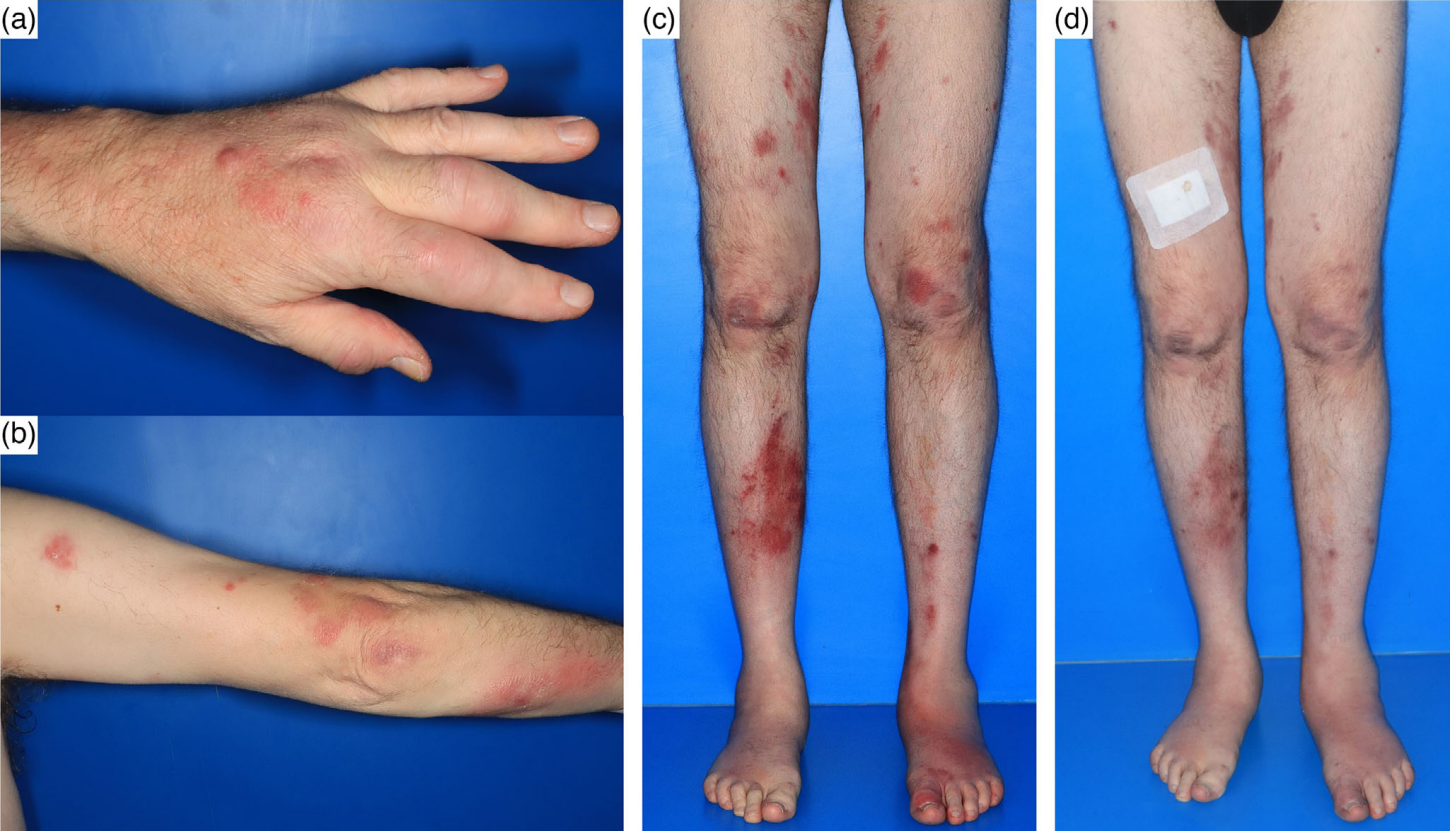
(a) Beginn der Nodi‐Ausbreitung an der Hand, mit der das Aquarium gereinigt wurde (Mai 2023). (b,c) Zunächst sporotrichoide Ausbreitung am Arm, anschließend Befall des gesamten Integuments (Mai 2023). (d) Deutliche Besserung des Hautbefundes etwa vier Wochen nach Therapiebeginn (Juni 2023).

Differenzialdiagnostisch wurden kutane Manifestationen des Morbus Crohn, unerwünschte Arzneimittelreaktionen oder Infektionen diskutiert. Eine Spindelbiopsie vom Oberschenkel zeigte in der H&E‐Färbung ein ausgeprägtes lymphohistiozytäres Infiltrat (Abbildung 2a,b). Immunhistochemisch fanden sich im Fettgewebe zahlreiche CD68‐positive Makrophagen (Abbildung 2c). Die Ziehl‐Neelsen‐Färbung blieb negativ (Abbildung 2d).

**ABBILDUNG 2:**
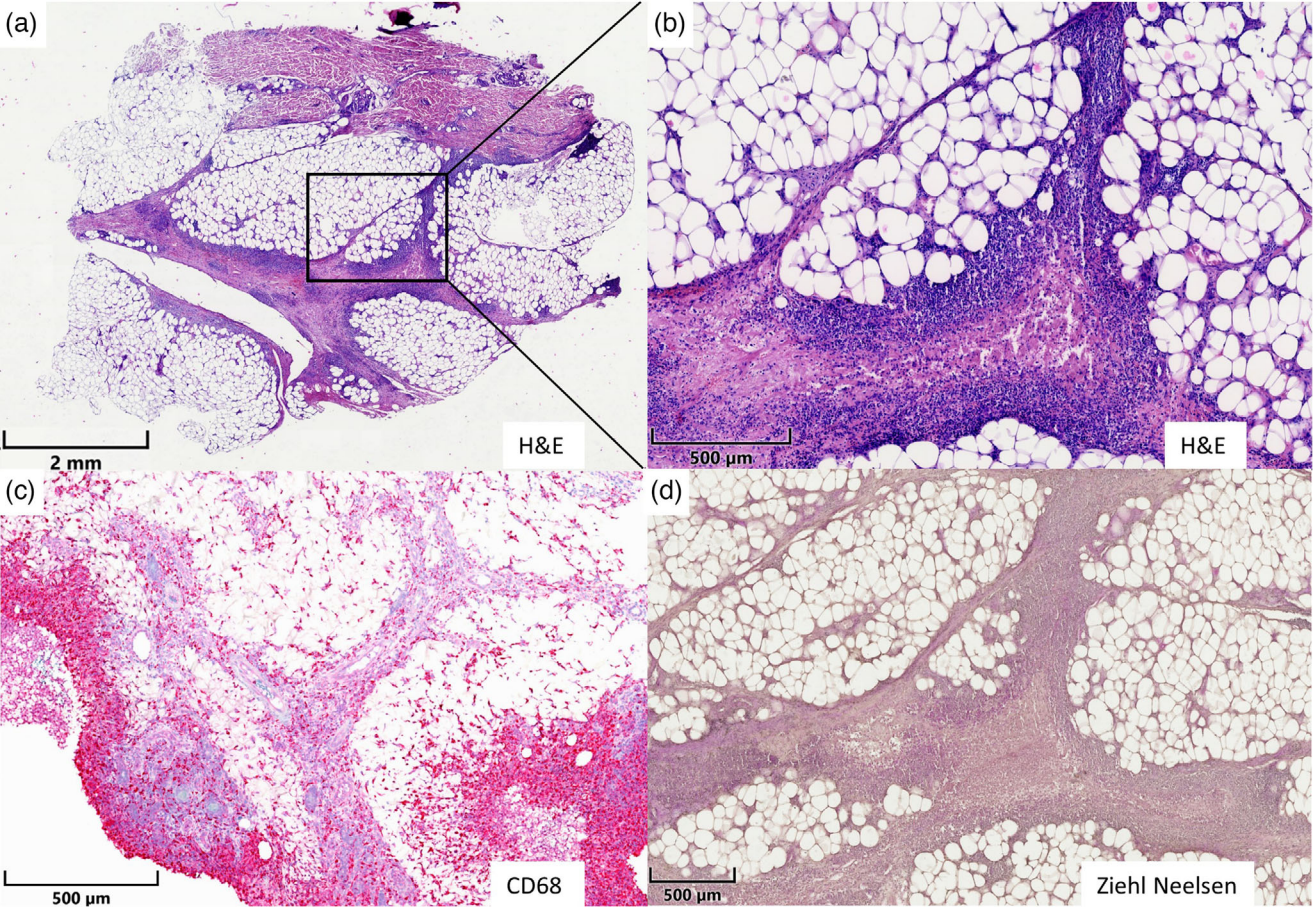
(a) Übersicht eines Biopsats vom Oberschenkel mit lymphozytärem Infiltrat in den Fettgewebssepten (H&E, Übersicht). (b) Vergrößerung mit gemischtzelligem, überwiegend lymphozytärem Infiltrat zwischen den Fettläppchen (H&E). (c): Nachweis zahlreicher CD68‐positiver Makrophagen (CD68). (d) Kein Nachweis säurefester Stäbchen (Ziehl‐Neelsen).

Mikrobiologisch gelang der Nachweis von M. marinum sowohl via PCR als auch kulturell. Im Flüssignährmedium blieb die Kultur negativ (Nachweisgrenze 10–100 KBE/ml). Auf einem Festnährmedium konnte M. marinum angezüchtet werden. Die Resistenztestung mittels Mikrodilution nach CLSI ergab eine MHK von 1,0 mg/L für Rifampicin (sensibel) und 2,0 mg/L für Ethambutol (keine Interpretation nach EUCAST 11.0).

Nach einer interdisziplinären Beratung wurde über einen Zeitraum von elf Monaten eine Tripletherapie mit Azithromycin, Ethambutol und Rifabutin durchgeführt. Diese Behandlung erforderte engmaschige Kontrollen: Azithromycin birgt das Risiko für gastrointestinale Beschwerden, Hörstörungen und QT‐Zeit‐Verlängerung; Ethambutol kann eine optische Neuritis auslösen; Rifabutin ist mit einer Hepatotoxizität, Uveitis und Myelosuppression assoziiert.^2^ Nach dem sich der klinische Zustand verbessert hatte, wurde Ethambutol abgesetzt und die Therapie dual mit Azithromycin und Rifabutin fortgeführt. Unter dieser Behandlung besserten sich sowohl die Hautveränderungen als auch die damit einhergehenden Schmerzen (Abbildung 1d). Im Verlauf kam es zu einer passageren Hörminderung sowie zu einer Hämaturie, vermutlich durch die Interaktion von Azithromycin mit einem DOAK, die aber beide reversibel waren.

Nicht tuberkulöse Mykobakterien (NTM) umfassen alle Mycobacterium‐Arten außer M. tuberculosis‐Komplex und M. leprae. Obwohl NTM in der Regel eine geringere Pathogenität aufweisen, können sie vielfältige Krankheitsbilder auslösen. Die Haut ist nach der Lunge der zweithäufigste Manifestationsort.^3^ Dabei handelt es sich um eine sehr heterogene Erregergruppe, bei der es teils erhebliche Unterschiede in der diagnostischen Abklärung, den Resistenzprofilen und den Therapieoptionen gibt. In diesem Kontext stellt Mycobacterium marinum einen besonderen Vertreter dar, der vor allem als typischer Erreger kutaner Infektionen bekannt ist, während andere NTM, wie M. fortuitum, M. kansasii oder M. ulcerans, unterschiedliche klinische Muster aufweisen. Mycobacterium fortuitum verursacht vor allem Abszesse und chronische Wunden, M. kansasii ist überwiegend mit pulmonalen Infektionen assoziiert, während M. ulcerans durch das Toxin Mykolakton ausgedehnte ulzerative Läsionen (Buruli‐Ulkus) hervorruft.^1^

Infektionen mit M. marinum entstehen typischerweise durch Kontakt von bereits vorher bestehenden Hautläsionen mit kontaminiertem Wasser. Klinisch treten purpurfarbene Papeln, Plaques oder Knoten bevorzugt an den Extremitäten auf, häufig in einer sporotrichoiden Anordnung.^4^ In unserem Fall kam es durch die Immunsuppression zu einer ungewöhnlich ausgeprägten Dissemination, sodass auch eine hämatogene Streuung angenommen werden muss.

Die Diagnosestellung erfolgt durch die Anamnese und Inspektion sowie durch die histopathologische Untersuchung einer Läsion und die Kultivierung beziehungsweise durch den Nachweis des Bakteriums mittels PCR. Histologisch zeigt sich in der Dermis ein charakteristischer granulomatöser und suppurativer Entzündungsprozess. Die Ziehl‐Neelsen‐Färbung blieb im vorliegenden Fall ohne Nachweis säurefester Stäbchen. Dies kommt bei M. marinum‐Infektionen angesichts der niedrigen Sensitivität dieser Methode häufig vor. Der diagnostische Goldstandard ist nach wie vor die kulturelle Anzucht mit anschließender Resistenzbestimmung. Dabei ist die Angabe der Verdachtsdiagnose bei der Anforderung entscheidend, um die für Mykobakterien erforderlichen besonderen Kulturbedingungen zu gewährleisten und falsch negative Ergebnisse zu vermeiden.^5^ Ergänzend können spezifische PCR‐Tests zur Identifikation und Differenzierung nicht‐tuberkulöser Mykobakterien (NTM) eingesetzt werden, die im Vergleich zu einer Kultur deutlich schnellere Ergebnisse liefern.^6^ Die Differenzialdiagnose umfasst die Sporotrichose, die Leishmaniose, Pilzinfektionen sowie granulomatöse Erkrankungen wie die Sarkoidose, Fremdkörperreaktionen, oder die Tuberkulose.^1^, ^6^

Die Wahl der Therapie beruht auf verschiedenen Faktoren, darunter der NTM‐Subtyp, das Ausmaß der Läsionen und der Immunstatus des Patienten. In leichten Fällen können Monotherapien mit Clarithromycin, Minocyclin, Doxycyclin oder Trimethoprim/Sulfamethoxazol wirksam sein. Schwere Fälle wie dieser erfordern häufig Kombinationstherapien, die beispielsweise Rifampicin, Ethambutol, Makrolide und Trimethoprim/Sulfamethoxazol beinhalten.^2^, ^4^ Obwohl einzelne Läsionen persistierten, kam es im vorliegenden Fall zu einer deutlichen klinischen Besserung, wodurch die Wirksamkeit der Therapie unterstrichen wurde.

Unter Adalimumab sind kutane Nebenwirkungen einschließlich infektiöser Komplikationen beschrieben. Neben makulopapulösen Ausschlägen oder psoriasiformen Läsionen kann die immunsuppressive Wirkung die Anfälligkeit für Infektionen mit atypischen Mykobakterien erhöhen.^7^ Ob im hier dargestellten Fall der Einsatz von Adalimumab die Infektion begünstigte, bleibt unklar, erscheint aber wahrscheinlich. Therapeutisch könnten alternative Immunsuppressiva wie IL‐23‐Blocker (z. B. Guselkumab) oder JAK‐Inhibitoren (z. B. Upadacitinib) erwogen werden,^8^, ^9^ da sie weniger häufig mit NTM‐Infektionen assoziiert sind.^10^

Dieser Fall verdeutlicht die Bedeutung einer frühzeitigen differenzialdiagnostischen Abklärung und einer gezielten mikrobiologischen Diagnostik für das Management atypischer Mykobakteriosen, insbesondere bei immunsupprimierten Patienten.

## DANKSAGUNG

Open access Veröffentlichung ermöglicht und organisiert durch Projekt DEAL.

## INTERESSENKONFLIKT

Keiner.
